# Serological Measures of Malaria Transmission in Haiti: Comparison of Longitudinal and Cross-Sectional Methods

**DOI:** 10.1371/journal.pone.0093684

**Published:** 2014-04-01

**Authors:** Benjamin F. Arnold, Jeffrey W. Priest, Katy L. Hamlin, Delynn M. Moss, John M. Colford Jr, Patrick J. Lammie

**Affiliations:** 1 Division of Epidemiology, School of Public Health, University of California, Berkeley, California, United States of America; 2 Division of Foodborne, Waterborne, and Environmental Diseases, Centers for Disease Control and Prevention, Atlanta, Georgia, United States of America; 3 Division of Parasitic Diseases and Malaria, Centers for Disease Control and Prevention, Atlanta, Georgia, United States of America; University of Melbourne, Australia

## Abstract

**Background:**

Efforts to monitor malaria transmission increasingly use cross-sectional surveys to estimate transmission intensity from seroprevalence data using malarial antibodies. To date, seroconversion rates estimated from cross-sectional surveys have not been compared to rates estimated in prospective cohorts. Our objective was to compare seroconversion rates estimated in a prospective cohort with those from a cross-sectional survey in a low-transmission population.

**Methods and Findings:**

The analysis included two studies from Haiti: a prospective cohort of 142 children ages ≤11 years followed for up to 9 years, and a concurrent cross-sectional survey of 383 individuals ages 0–90 years old. From all individuals, we analyzed 1,154 blood spot specimens for the malaria antibody MSP-1_19_ using a multiplex bead antigen assay. We classified individuals as positive for malaria using a cutoff derived from the mean plus 3 standard deviations in antibody responses from a negative control set of unexposed individuals. We estimated prospective seroconversion rates from the longitudinal cohort based on 13 incident seroconversions among 646 person-years at risk. We also estimated seroconversion rates from the cross-sectional survey using a reversible catalytic model fit with maximum likelihood. We found the two approaches provided consistent results: the seroconversion rate for ages ≤11 years was 0.020 (0.010, 0.032) estimated prospectively versus 0.023 (0.001, 0.052) in the cross-sectional survey.

**Conclusions:**

The estimation of seroconversion rates using cross-sectional data is a widespread and generalizable problem for many infectious diseases that can be measured using antibody titers. The consistency between these two estimates lends credibility to model-based estimates of malaria seroconversion rates using cross-sectional surveys. This study also demonstrates the utility of including malaria antibody measures in multiplex assays alongside targets for vaccine coverage and other neglected tropical diseases, which together could comprise an integrated, large-scale serological surveillance platform.

## Introduction

Efforts to monitor malaria transmission to inform control strategies increasingly use cross-sectional surveys to estimate transmission intensity from seroprevalence data based on malaria antibodies [1]–[8]. The approach has gained popularity because malaria antibody levels can be measured from dried blood spots [9], which are relatively easy to collect in the field in cross-sectional surveys, and this approach to estimating transmission intensity is far more cost effective and simple compared to alternative methods such as estimating the entomologic inoculation rate. Another major advantage of the approach compared to other low-cost methods, such as rapid diagnostic tests, is that the longevity of antibody responses makes them potentially more sensitive and informative measure of transmission in low-transmission environments [2]. A potential disadvantage of using antibody measures to estimate transmission intensity is that some antibody responses could saturate at a low transmission intensity, thus providing less useful information as a monitoring tool as transmission declines [1]. Nevertheless, serological measures of malaria infection have been proposed as a preferred diagnostic to measure community level transmission in the pre-elimination and elimination phases of malaria control [10].

Investigators have estimated malaria transmission intensity from cross-sectional prevalence surveys using seroconversion rates estimated with a reversible catalytic model [2]. Previous validation efforts have shown that the entomological inoculation rate – the main measure of transmission intensity – is strongly correlated with seroconversion rates estimated with a model fitted to cross-sectional data [1], [2]. However, to our knowledge, this model-based approach has not been validated with incident seroconversion rates measured prospectively in a longitudinal cohort. Given the increasing use of cross-sectional serological surveys to monitor malaria transmission, ensuring that model-based seroconversion rates estimated from cross-sectional surveys are consistent with rates estimated in prospective cohorts is an important and necessary step to validate the approach.

The objective of this study was to estimate the malaria seroconversion rate using antibody measures against merozoite surface protein-1_19_ (MSP-1_19_) from incident seroconversions measured in a longitudinal cohort of Haitian children ages 0–11 years old, and compare it to the rate estimated with a reversible catalytic model fit to a cross-sectional survey of Haitians aged 0–90 years old. Since the longitudinal data provide a direct measure of the seroconversion rate, a comparison of estimates from the two approaches provides an important check of the model's consistency as currently applied in low-transmission settings.

## Materials and Methods

### Study Population and sample collection

Study populations were set up initially to monitor transmission of lymphatic filariasis (LF) in a setting of intense LF transmission. Both longitudinal and cross sectional studies were carried out in the coastal plain near Léogâne, Haiti, where up to half of the population was infected with *Wuchereria bancrofti* [11]. Longitudinal samples were collected as part of a study of the role of maternal LF infection on acquisition of LF as previously described [12], [13]. A total of 142 children were enrolled between the ages of 1 month and 6 years on a rolling basis and followed up at 6–12 month intervals from 1991–1999 to monitor LF parasitologically and serologically. Children were followed for up to 9 years and each child was measured approximately once per year. Finger prick blood samples were collected from the study children. Additional details of the cohort have been previously reported [13]. Cross-sectional blood samples were collected by finger prick in 1998 from 383 individuals in the community of Miton (approximately 5 km from the communities included in the longitudinal study) as part of an intervention trial to investigate the impact of salt fortified with diethylcarbamazine on LF [14]. Donor ages ranged from 2 weeks to 90 years.

### Ethics Statement

The protocols for both studies were reviewed and approved by the Centers for Disease Control and Prevention's Institutional Review Board and the Ethical Committee of St. Croix Hospital (Léogâne, Haiti). After explaining the purpose of the study in Creole, individuals were asked to provide verbal consent to participate in the research. The human subjects review boards approved the verbal consent process due to low literacy rates in the study communities. In cases of longitudinal follow-up, the study team documented consent at each study visit. Mothers provided consent for young children, and children 7 years or older provided assent. Consent forms included specific permission to share specimens and to test the samples for other infectious diseases.

### Malaria antibody measurement and determination of seroconversion events

A recombinant GST/MSP-1_19_ fusion protein cloned from *P. falciparum* isolate 3D7 (kindly provided by C.W. Kauth and H. Bujard, Heidelberg University, Germany) was coupled to SeroMap beads (Luminex Corp., Austin, TX) in phosphate-buffered saline (pH 7.2) as previously described [13], [15]. A total of 120 μg protein was coupled to 12.5×10^6^ beads. The MSP-1_19_ assay was included as part of a 28-plex panel for the longitudinal survey [13] and as part of a 16-plex panel for the cross-sectional survey. Multiplex assays were conducted using sera diluted in a polyvinyl alcohol- and polyvinylpyrrolidone-containing buffer (1∶400) and the biotinylated monoclonal anti-IgG and IgG4 antibodies previously described [13]. Data were reported as the average median fluorescent intensity *minus* background (MFI-bg) for the duplicate wells. The magnitude of the fluorescent response (reported in MFI – bg units) is proportional to the amount of antigen-specific IgG antibody present in the sample.

We used adult US citizens with no history of foreign travel as an unexposed population for antibody cutoff values to classify individuals as seropositive. The mean +3*SD for the MSP-1_19_ antibody response was calculated from the logged values of the negative control antibody responses. Because samples from the longitudinal study and the cross-sectional study were run with two different bead lots, two different cutoff values were used: a cutoff of 365 MFI-bg units was calculated from 63 negative control sera for the longitudinal study bead set, and a cutoff of 477 MFI-bg units was calculated from 70 negative control samples for the cross-sectional study bead set.

Four children <6 months in the longitudinal cohort and one child age 2 months in the cross-sectional study had evidence of maternal antibodies to multiple antigens (including malaria) in the multiplex panel; we classified them as seronegative in their first year of life for the analysis. For children who were classified as seropositive during follow-up we plotted their antibody responses to identify those who were incident cases versus those who were positive at their first visit.

### Statistical methods

#### Estimation of seroconversion rates with longitudinal data

We estimated age-specific seroprevalence in the longitudinal cohort by combining measurements into one-year to three-year age groups that included enough measurements to estimate each prevalence with reasonable precision. We also estimated the seroconversion and seroreversion rates using incident conversions and reversions divided by the person time at risk over follow-up [16]. Person time at risk for seroconversion was estimated by summing the person time for periods where children were seronegative at the beginning of the period. We estimated the seroreversion rate in an analogous fashion, but used periods where children began the period as seropositive. To estimate variability of the prevalence and rate estimates, we bootstrapped the dataset with 10,000 replications, resampling children with replacement, and used the 2.5 and 97.5 percentiles of the bootstrapped sampling distribution to construct 95% confidence intervals.

With short periods between measurements such as days, the approach to estimating the rates directly is unbiased [17], but for longer periods between measurements similar to those used in this study (typically one year) there is some concern that this approach could under-estimate the seroconversion rate; the rationale is that it is possible for a child to become infected and recover between measurements, and those cases would not be detected by the surveillance [16]. For this reason, previous longitudinal studies with measurements separated by weeks or months have also estimated malaria incidence and recovery rates using a reversible catalytic model that attempts to account for the possibility of multiple infections [16], [18]. Since the antibody response to malarial antigens can last for years in the presence of single- or multiple infections [19], we would expect seroconversion rates estimated from incident cases and from a catalytic model to coincide; we compared the two approaches as an internal validity check. Text S1 Statistical Details includes details of the reversible catalytic model used in the analysis. All analyses were conducted using R version 2.15.2 (www.R-project.org).

#### Estimation of seroconversion rates with cross-sectional data

With cross-sectional data, direct information about seroconversion and seroreversion is unknown since the same individual is not observed at two points in time. Instead, there is only current status information for an individual at one point in time when they are a particular age. The reversible catalytic model for incidence data can be fit to prevalence data with a simplification where exposure time is measured by age in years [1], [2]. As with the longitudinal version of the model, the model parameters estimate seroconversion and seroreversion rates. Text S1 Statistical Details includes model details. We fit the age-specific seroconversion model to the cross-sectional dataset using all individuals aged 0–90 years old, and separately for individuals ages 0–11 years old for a direct comparison with the longitudinal cohort.

## Results

### Population characteristics

**Figure 1:**
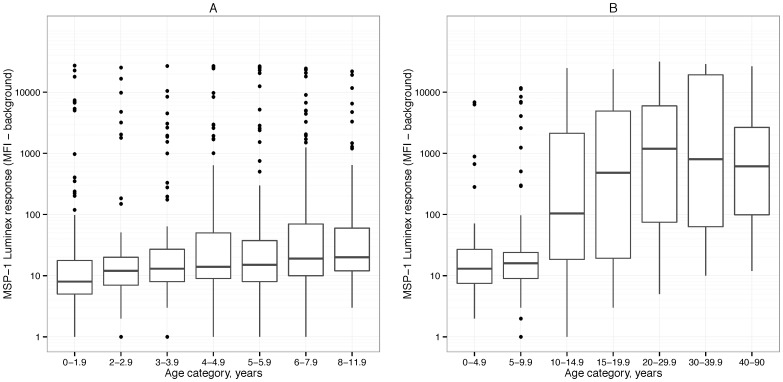
MSP-1_19_ antibody optical density responses for different age categories. Panel A includes antibody responses from the longitudinal study in Léogâne, Haiti, 1991–1999. Panel B includes antibody responses from the cross-sectional survey in Miton, Haiti, 1998.

Among the 142 children enrolled in the longitudinal study in Leogane, each child was followed for an average of 5.1 years (range  = 0.5, 9.1). The study included 771 total antibody measurements, and the average number of measurements per child was 5.4 (range  = 2, 9). The 383 individuals enrolled in the cross-sectional survey in Miton ranged in age from two weeks to 90 years old. Antibody response increased with age in both samples (Figure 1).

### Seroprevalence estimates

Table 1 summarizes the seroprevalence measured in the longitudinal cohort. Seroprevalence increased in the first years of life, from 5% among children <2 years old to 17% for children 6–8 years old. Table 2 summarizes seroprevalence in the cross-sectional study for different age categories, which showed a similar increase in seroprevalence that plateaued by age 20 at over 50%. The seroprevalence estimates for children ≤11 years old were comparable in the two samples (11% 95% CI: [7%, 16%] in the longitudinal study v. 14% [9%, 20%] in the cross-sectional study).

**Table 1:** Age-specific seroprevalence estimates based on the MSP-1_19_ antibody measured in 142 children followed longitudinally in Léogâne, Haiti 1991–1999.

Age Category (years)	Median Age (years)	N	%	95% CI
[0–2)	1.0	127	5	(1, 9)
[2–3)	2.5	98	7	(2, 14)
[3–4)	3.5	114	10	(4, 16)
[4–5)	4.6	103	15	(7, 23)
[5–6)	5.4	115	12	(6, 20)
[6–8)	6.8	125	17	(8, 26)
[8–11.9]	9.1	89	12	(3, 24)
All ages [0–11.9]	4.5	771	11	(7, 16)

N is number of measurements.

CI: Confidence Interval.

**Table 2:** Age-specific seroprevalence estimates based on the MSP-1_19_ antibody in Miton, Haiti in 1998.

Age Category (years)	Median Age (years)	N	%	95% CI
[0–5)	2	51	6	(1, 16)
[5–10)	7	67	13	(6, 24)
[10–15)	12	82	34	(24, 45)
[15–20)	16	43	49	(33, 65)
[20–30)	24	51	57	(42, 71)
[30–40)	33	32	53	(35, 71)
[40–90]	50	57	54	(41, 68)
All ages [0–90]	14	383	36	(31, 41)
Ages [0–11]	7	157	14	(9, 20)

N is number of individuals.

CI: Confidence Interval.

### Seroconversion rate estimates

**Figure 2:**
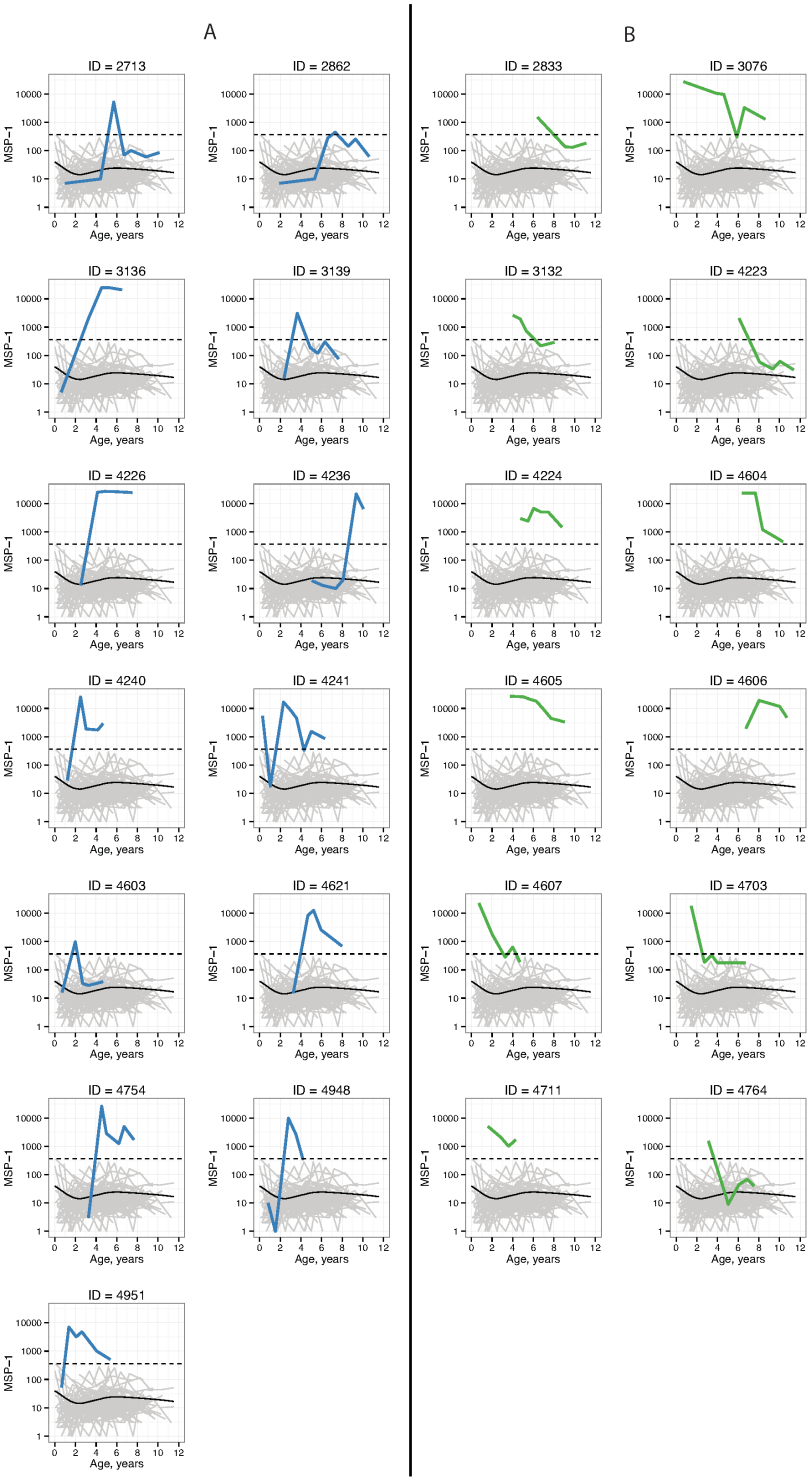
MSP-1_19_ antibody optical density profiles for children in the Léogâne, Haiti longitudinal study, 1991–1999. Panel A includes individuals with incident seroconversions, and panel B includes those who were seropositive at their first measurement. The dashed line marks the cutoff value (365) used to determine seropositive antibody levels. The light grey lines plot antibody profiles for seronegative children, and the solid black line in each plot is a loess smoother over the seronegative children antibody levels.

In the longitudinal study, 25 children were identified as seropositive for malaria during follow-up. Of these, 13 were incident seroconversions and 12 had seroconverted by their first measurement (Figure 2). No children showed a pattern of multiple infections based on their MSP-1_19_ antibody levels. Over the 9 years of follow-up, there were 558 periods where children began periods as seronegative corresponding to 646.185 person years; there were 71 periods where children began periods as seropositive corresponding to 71.844 person years. There were 13 incident seroconversions (rate  = 0.020 per year) and 11 incident seroreversions (rate  = 0.153 per year).

**Figure 3:**
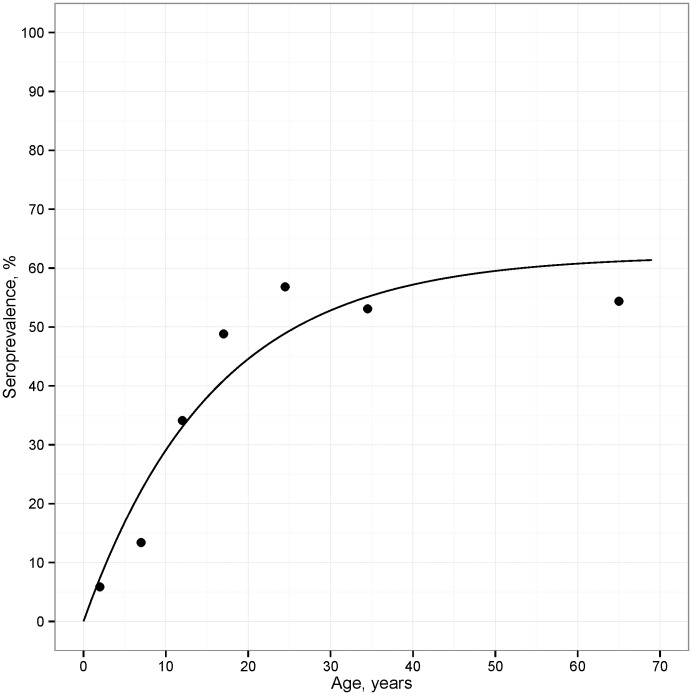
Seroprevalence estimates from the cross-sectional survey in Miton, Haiti, 1998. Seroprevalence estimates for the age categories in Table 2 (points) are plotted at the midpoint of the age categories, and the line in the plot is the predicted prevalence from the reversible catalytic model.

Table 3 compares seroconversion and seroreversion rate estimates from the longitudinal study with those from the cross-sectional study. Seroconversion rates estimated among children ages ≤11 years were highly comparable using the two approaches (seroconversion rate estimated from incidence data  = 0.020 [0.010, 0.032]; estimated from cross-sectional data  = 0.023 [0.001, 0.052]; Table 3). The seroconversion rate estimated with the model across all ages in the cross-sectional sample was 0.039 (0.027, 0.052), and the model provided a reasonable fit to the seroprevalence data (Figure 3). The seroreversion rate could not be estimated accurately with either approach due to the small number of individuals in the studies who were seropositive.

**Table 3:** Comparison of seroconversion and reversion rates calculated from different study cohorts and different estimation approaches.

Study	Estimation approach	Seroconversion rate (95% CI)	Seroreversion rate (95% CI)
Longitudinal cohort (Léogâne)	Incident cases, ages ≤11 y	0.020 (0.010, 0.032)	0.153 (0.073, 0.266)
	RC model, ages ≤11 y*	0.021 (0.001, 0.096)	0.163 (0.001, 0.729)
Cross-sectional survey (Miton)	RC model, ages ≤11 y*	0.023 (0.001, 0.052)	0.001 (0.001, 0.255)
	RC model, all ages	0.039 (0.027, 0.052)	0.024 (0.005, 0.043)

CI: Confidence Interval; RC: Reversible Catalytic model.

* Lower 95% confidence intervals truncated at the lower bound of possible parameter values (0.001).

## Discussion

In this analysis of two separate studies from Haiti we found that the seroconversion rate for the MSP-1_19_ malaria antibody was very similar when estimated from incident seroconversions in a longitudinal cohort and from a model fit to cross-sectional seroprevalence data. This finding is important because it provides a direct validation of the use of cross-sectional malaria seroprevalence data to estimate seroconversion rates in low-transmission settings. Earlier analyses demonstrated that the seroconversion rate measured from cross-sectional surveys was strongly associated with the entomological inoculation rate [1], [2]; our finding of concordance of seroconversion rates estimated from separate longitudinal and cross-sectional studies in the same low-transmission region lends further credibility to this approach, and underscores its potential utility in large-scale, cross-sectional surveillance activities.

The estimation of seroconversion rates using cross-sectional data is a widespread and generalizable problem for many infectious diseases. Although the model used to estimate malaria seroconversion rates from cross-sectional surveys is an extreme simplification of a complex immunological process, numerous field studies including the present study have found that the model fits the data well [1]–[8]. Simple, parsimonious models such as the one used in this analysis should be favored over more complex models as long as the simple approach provides a reasonable approximation to the observed data [20]. A series of recent studies have added one layer of model complexity to allow for multiple seroconversion rates to be estimated for different age groups [3], [5]–[7]. A motivation for estimating separate rates in different age groups is to allow for changes in transmission due to intervention. In the present study, there was no well-defined intervention that would have abruptly changed transmission, and a single seroconversion rate over all ages provided a good fit to the seroprevalence data (Figure 3). Nevertheless, the seroconversion rate estimated for children ages 0–11 years was lower than the rate estimated using data from all ages in the cross-sectional survey, suggesting potentially reduced transmission in the younger cohort. Text S1 Statistical Details includes model results from the cross-sectional survey that allowed for two seroconversion rates with an age breakpoint chosen to maximize the overall likelihood [3]; consistent with the primary analysis, the more complex model estimated a lower seroconversion rate among younger individuals compared to older individuals, which is consistent with a secular trend of reduced transmission in this population. Based on these data, it would appear that transmission of malaria in Haiti is relatively low and, at least anecdotally, it appears that transmission has either declined in recent years or is less intense at younger ages. Potential explanations for a secular decline are not clear as there were no systematic control activities during the time period when samples were collected. Importantly, we found no evidence for antibody saturation using MSP-1_19_, which underscores its utility as a sensitive surveillance tool in low-transmission settings.

More recently, Bretscher et al. [21] used a different approach based on a Bayesian Hidden Markov Model to estimate seroconversion and reversion rates from a longitudinal cohort in Indonesia. Rather than classify individual children as seropositive or negative at each point in time, as we did in the present analysis, the approach assigns a probability to each measurement of the likelihood that the individual is infected. The rationale that Bretscher et al. used to justify using a more complex Hidden Markov Model is twofold: (i) individual seroconversion events are lost when converting continuous antibody measurements to prevalence in different age groups, and (ii) that antibody levels are inherently noisy and imposing a fixed cutoff may lead to false conversion and reversion events.

Our results suggest that the simpler calculation of seroconversion rates directly from incident seroconversions is a viable alternative to a more complex, Hidden Markov Model approach. First, as we have demonstrated there is no need to collapse individual information into age categories and group level seroprevalence to estimate seroconversion rates with longitudinal data – rates can be estimated directly from incident seroconversions [16]–[18]. However, the issue of antibody variability around a fixed cutoff value is a valid concern. In the present study, the majority of seropositive antibody measures were far beyond the threshold but there were instances where antibody levels oscillated around the cutoff value (e.g., IDs 3132, 4607, 4703; Figure 2). We used individual antibody profile plots (Figure 2) and scientific judgment to rule out non-incident conversions in the rate calculations. This was feasible for a study with 25 seropositive children and 13 incident seroconversions and would be a reasonable approach for similarly sized studies (e.g., Bretscher et al. observed just 3 incident seroconversions in their study [21]). In a study with a very large number of seroconversions then it should be possible to develop a simple algorithmic approach that identifies incident seroconversions for a given antibody based on a defined period spent seronegative before the conversion. Future studies could also consider the use of quantitative antibody responses to measure malaria transmission. The use of quantitative antibody responses would avoid the problem of choosing a cutoff for classifying seropositive samples and would potentially retain more information in the analysis. The methods required to estimate malaria transmission intensity from quantitative antibody responses is a potential area for future research.

Serum samples from these communities were tested by multiplex as part of a study of risk factors for acquisition of LF; MSP-1_19_ was included in the multiplex, along with antigens from enteric pathogens and vaccine-preventable diseases to better understand the public health context in these communities and the potential interactions between LF and other infections. The current results illustrate the potential of this approach to capture seroincidence data for infections beyond those that were the initial focus of the study. Less clear at this point is the extent to which reversible catalytic models might be successfully applied to the other infections we monitored. These efforts are the focus on ongoing efforts in our labs. Independent of whether or not reversible catalytic models can be applied generally as measures of transmission intensity, multiplex serologic assays represent a powerful tool for capturing useful public health data with simple, dried blood spot surveys.

A limitation of the analysis was that only 25 children were classified as seropositive in the longitudinal study, which meant that it was impossible to estimate age-specific seroconversion rates and it was also impossible to estimate the seroreversion rate with precision. The wide confidence intervals and variation in point estimates for the seroreversion rate in analyses limited to children ≤11 years reflect the lack of information needed to accurately estimate that parameter (Table 3). Although the estimate of the seroreversion rate from the catalytic conversion model fit to cross-sectional data over all ages was more precise, we expect imprecise estimation of seroreversion rates to be a common problem for studies in low-transmission settings because of the relatively low prevalence of infection – this limitation was pointed out in an early description of the approach [2]. However, if the main parameter of interest is the seroconversion rate, then this study suggests that it can be estimated with reasonable precision despite limited information about seroreversion. Even if a study's main focus is mainly the seroconversion rate, estimating differences in that rate – either by age, over time, or between groups – requires that the study collect sufficient observations in each group to accurately estimate separate rates.

These studies were not designed to follow malaria specifically and we have no parasitologic data that would confirm that children were, in fact, malaria infected. That limitation notwithstanding, not all malaria infections may manifest as patent parasitemia, so serological measures of infection may be a more accurate representation of the true level of transmission in the population – particularly in areas of low transmission [2]. It is also important to point out that the longitudinal and cross-sectional specimens were not collected from the same community; however, these communities are located with 5 km of each other and are similar in terms of ethnicity, genetic heritage, house structures, local environments and socioeconomic status. Although MSP-1_19_ antibody responses are considered to be sensitive and specific measures of malaria infection, use of multiple malaria antigens would improve the sensitivity of case detection. We estimate that use of a single antigen would capture at least 75% of incident infections (Priest et al., unpublished observations), so our estimates of infection prevalence should be reasonably accurate. Inclusion of additional malaria antigens in future studies, a straightforward approach with the multiplex, will add to the value of these analyses.

## Conclusion

Our finding of close agreement between malaria seroconversion rates estimated in a prospective cohort study with those estimated using a reversible catalytic model fit to cross-sectional prevalence data lends additional credibility to the use of cross-sectional, serological surveys to monitor malaria transmission in low-transmission settings. These results demonstrate the utility of including malaria antibody measures in multiplex assays alongside targets for vaccine coverage and other neglected tropical diseases, which together could comprise an integrated, large-scale surveillance platform.

## Supporting Information

Text S1 **Statistical Details.** This supporting information includes details about the statistical models used in the analysis as well as additional seroconversion rate estimates using a two rate model.
(PDF)
