# Supplementary material for: Serological Measures of Malaria Transmission in Haiti: Comparison of Longitudinal and Cross-Sectional Methods
Source: PLoS One. 2014 Apr 1;9(4):e93684. doi: 10.1371/journal.pone.0093684 (PMC3972142; doi:10.1371/journal.pone.0093684)
Supplement: Text S1 — Statistical Details. This supporting information includes details about the statistical models used in the analysis as well as additional seroconversion rate estimates using a two rate model. (PDF) [file pone.0093684.s001.pdf]

Text S1 Statistical Details

Serological measures of malaria transmission in Haiti: comparison of longitudinal and cross-sectional methods

Benjamin F. Arnold, Jeffrey W. Priest, Katy L. Hamlin, Delynn M. Moss, John M. Colford, Jr., and Patrick J. Lammie

Reversible catalytic model for longitudinal data

The original model proposed by Bekessy et al. [1] assumes that infection follows a Markov process where an individual in period t can either be positive or negative. Their status can either stay the same in time $t+1$ or they can change states (either from positive to negative, or vice versa). The model assumes that the probability of converting between states follows a binomial process and that the process has no "memory" – the transition probabilities between t and $t+1$ use no information for time periods before t. After solving a system of differential equations Bekessy et al. [1] show that under the model, the probability of infection in period t is:

$$P_I(t) = \frac{\lambda}{\lambda+\rho}\left[1-\exp(-(\lambda+\rho)t)\right] \quad (1)$$

and the probability of recovery in period t is:

$$P_R(t) = \frac{\rho}{\lambda+\rho}\left[1-\exp(-(\lambda+\rho)t)\right] \quad (2)$$

In both probabilities, λ is the incidence rate and ρ is the recovery rate. With serological antibody data, the incidence rate is estimated by seroconversion, and recovery by seroreversion. The parameters can be estimated empirically using maximum likelihood. For a binomial random variable $Y_{i,t}$ that indicates infection status for individual i at time t, the part of likelihood function that depends the probabilities of infection and recovery

under the model is:

$$\begin{aligned} L(\theta) &= \prod_{i=1}^{n}\prod_{t=1}^{T_i} P(Y_{i,t}|Y_{i,t-1}) \\ &= \prod_{i=1}^{n}\prod_{t=1}^{T_i} P_I(t)^{Y_{i,t}(0,1)}[1-P_I(t)]^{Y_{i,t}(0,0)} \cdot P_R(t)^{Y_{i,t}(1,0)}[1-P_R(t)]^{Y_{i,t}(1,1)} \end{aligned} \quad (3)$$

where $P_I(t)$ and $P_R(t)$ are the transition probabilities in equations 1 and 2 and $Y_{i,t}(s_t, s_{t+1})$ are indicator variables for the status of individual i (0=negative, 1=positive) at the beginning and end of the period. The log likelihood is:

$$\begin{aligned} \ell(\theta) &= \sum_{i=1}^{n}\sum_{t=1}^{T_i} Y_{i,t}(0,1)\log P_I(t) + Y_{i,t}(0,0)\log[1-P_I(t)] + \\ &\qquad Y_{i,t}(1,0)\log P_R(t) + Y_{i,t}(1,1)\log[1-P_R(t)] \end{aligned} \quad (4)$$

We estimated the seroconversion and seroreversion rates by maximizing the likelihood in equation 4 given the observed data. The R function used for the likelihood is at the end of this appendix. To estimate the variance of prevalence estimates and model-based seroconversion and seroreversion rates in the longitudinal cohort, we used a clustered bootstrapped approach where we resampled individuals with replacement 10,000 times, and in each iteration estimated the parameters using maximum likelihood. This preserved the within-child correlation in outcome measurements. The SD of the bootstrap distribution was used to estimate the SE of the parameters, and the 2.5 and 97.5 percentiles of the bootstrap distribution were used as the 95% confidence intervals.

Estimating seroconversion rates using cross-sectional data

With cross-sectional data, direct information about seroconversion and seroreversion is unknown since we do not observe the same individual at two points in time and cannot identify incident cases. Instead, we only have current status information for an individual at one point in time when they are a particular age. Investigators have used a model similar to the one described in the last section to model the age-specific probability of

infection [2, 3]. The probability of infection at age a is modeled as:

$$P(a) = \frac{\lambda}{\lambda + \rho} [1 - \exp(-(\lambda + \rho)a)] \tag{5}$$

Note that this is similar to equation 1, but rather than modeling incident seroconversions and using person-time in a period of observation (t), here the equation summarizes the prevalence at age (a). Since there is only one probability in the model, the log likelihood function is identical in form to equation 4 but without the last two terms.

$$\ell(\theta) = \sum_{i=1}^{n} Y_i \log P(a) + (1 - Y_i) \log[1 - P(a)] \tag{6}$$

where Y_i is an indicator variable equal to 1 if individual i is positive and $P(a)$ is the probability of infection described in equation 5. Estimates of $\hat{\lambda}$ and $\hat{\rho}$ in a population can be obtained by maximizing the likelihood function given the observed status Y_i.

We fit the age-specific prevalence model to the cross-sectional dataset using all individuals aged 0 - 90 years old, and separately for individuals ages 0-11 years old for a direct comparison with the longitudinal data.

Estimating 2 seroconversion rates using cross-sectional data

One extension to the basic model for cross sectional data is to allow the seroconversion rate to differ for different age groups, and investigators have chosen the breakpoint for allowing the rate to change at a point that maximizes the overall likelihood (identified with a profile likelihood plot) [4–7]. We used a similar approach, but used cross-validation to select the break point in age to avoid the potential for choosing a break point that overfitted the data [8, 9]. If the entire dataset were used to choose a breakpoint parameter that maximizes the likelihood, it is possible for the model to overfit the data. Instead, choosing model parameters such as the breakpoint age for where the seroconversion rate should change using V-fold cross validation should be far less sensitive to overfitting. Hastie et al. [8] provide details of V-fold cross-validation; in brief, the approach randomly splits the data into V roughly equal sized parts. For the vth part, the model is fit on the other $V-1$ parts of the data, and loss (prediction error) is calculated on the vth part that

is left out as the test set. The process is repeated for each of the V splits, and the loss function is averaged over the V splits. In this particular application, we used V=5 data splits in the cross validation and the negative log likelihood as the loss function. Finally, we fit a single seroreversion rate across all ages in the survey (not separately for each age group). The standard maximum likelihood approach and cross validation approach produced highly comparable loss functions in this dataset (Figure S1). Both functions were maximized with a split at age 8 years. Figure S2 plots the model predictions allowing for two seroconversion rates along with observed seroprevalence.

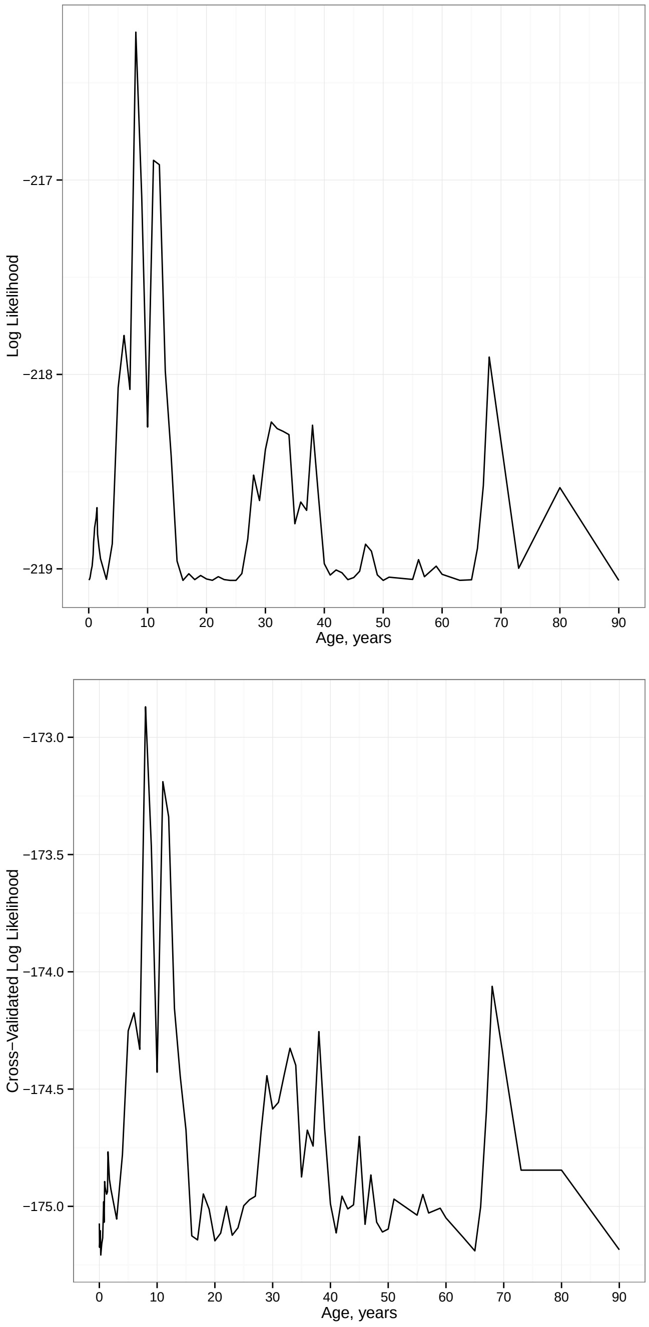

Figure S1: Profile log likelihood plot (top panel) and cross-validated log likelihood plot (bottom panel) allowing seroconversion rates to vary at different age splits

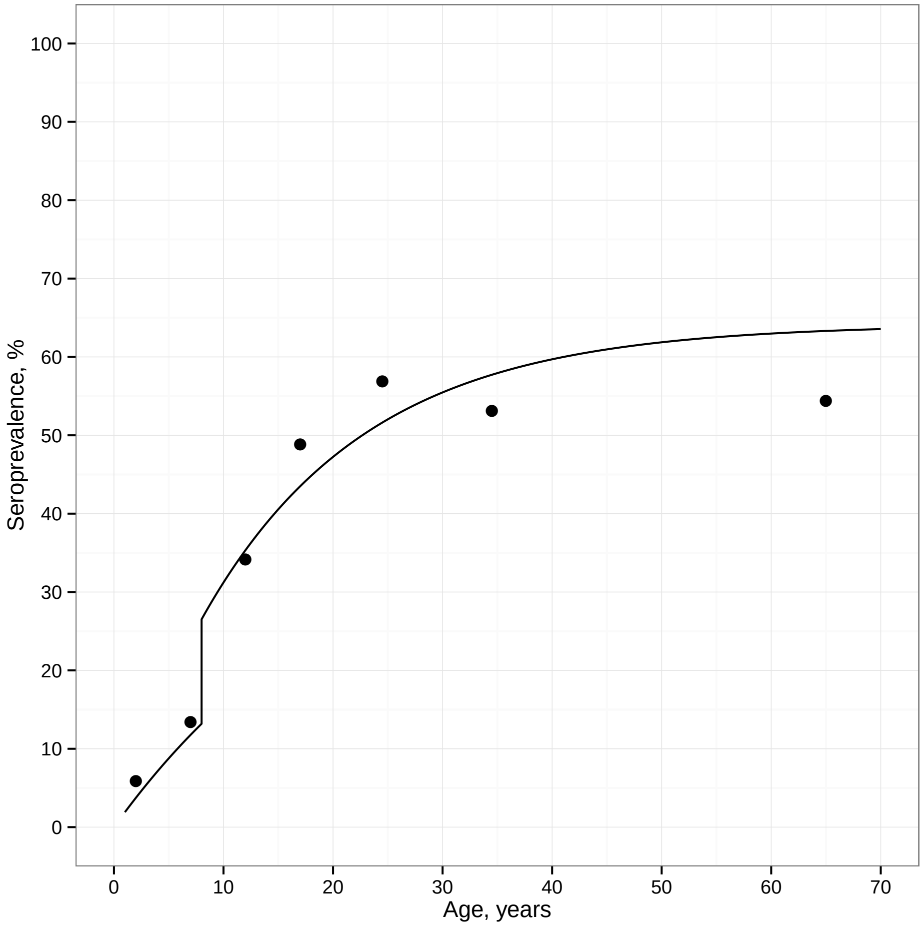

Figure S2: Seroprevalence estimates from the cross-sectional survey plotted at the mid-point of age categories in Table 2 (points) and predicted prevalence from the reverse catalytic model (line). The model fitted to the data allowed for different seroconversion rates below and above age 8 (chosen through cross-validation). For ages ≤ 8 years, the seroconversion rate was 0.020 (0.006, 0.033) and the seroconversion rate for ages $>$8 years was 0.043 (0.034, 0.051). The model estimated a single seroreversion rate over the entire population: 0.024 (0.005, 0.043).

R functions used for maximum likelihood estimation

R function for maximum likelihood estimation of a reversible catalytic model fit to cross-sectional data, using an example from Williams [10].

```
Lccm1 <- function(theta,data) {
# theta : vector of length two, including h, r (parameters to be maximized)
#         h = seroconversion rate, r = seroreversion rate
#        (to maximize over a single parameter, e.g. h, replace theta with
#         separate args for h and r and pass an r value to the function)
# data  : data frame with columns = age / n / k
h <- rep(theta[1],nrow(data))
r <- rep(theta[2],nrow(data))
t <- data[,1]
n <- data[,2]
k <- data[,3]
p <- h/(r+h)*(1-exp(-(r+h)*t))
# negative log likelihood function
sum( - (k)*log(p) - (n-k)*log(1-p) )
}
# data from Table 2 of Williams 1994
dog <- data.frame(
age=c(3.71,10.76,21.00,38.89,64.07,106.82),
n=c(100,74,114,110,55,27),
k=c(14,30,63,59,37,14)
)
# MLE solution
dogll <- optim(c(0.1,0.1),fn=Lccm1,data=dog,hessian=TRUE)
```

R function for maximum likelihood estimation of a reversible catalytic model fit to longitudinal data, following Annex 1 of Bekessy [1].

```
Lccm2 <- function(theta,data) {
# theta : vector of length two, including h, r (parameters to be maximized)
#         h = seroconversion rate, r = seroreversion rate
# data  : data frame with cols = interval of the period t to t+1
#     / status at time t / status at time t+1
h <- rep(theta[1],nrow(data))
r <- rep(theta[2],nrow(data))
t <- data[,1]
St  <- data[,2]
Stt  <- data[,3]
Nn  <- ifelse(St==0,1,0)
Nnp <- ifelse(St==0 & Stt==1,1,0)
Np  <- ifelse(St==1,1,0)
Npn <- ifelse(St==1 & Stt==0,1,0)
ph <- h/(r+h)*(1-exp(-(r+h)*t))
pr <- r/(r+h)*(1-exp(-(r+h)*t))
# negative log likelihood function
sum( -(Npn)*log(pr) - (Np-Npn)*log(1-pr) - (Nnp)*log(ph) - (Nn-Nnp)*log(1-ph) )
}
```

References

[1] Bekessy A, Molineaux L, Storey J. Estimation of incidence and recovery rates of Plasmodium falciparum parasitaemia from longitudinal data. Bull World Health Organ. 1976;54(6):685–693.

[2] Drakeley CJ, Corran PH, Coleman PG, Tongren JE, McDonald SLR, Carneiro I, et al. Estimating medium- and long-term trends in malaria transmission by using serological markers of malaria exposure. Proc Natl Acad Sci U S A. 2005 Apr;102(14):5108–5113. Available from: http://dx.doi.org/10.1073/pnas.0408725102.

[3] Corran P, Coleman P, Riley E, Drakeley C. Serology: a robust indicator of malaria transmission intensity? Trends Parasitol. 2007 Dec;23(12):575–582. Available from: http://dx.doi.org/10.1016/j.pt.2007.08.023.

[4] Stewart L, Gosling R, Griffin J, Gesase S, Campo J, Hashim R, et al. Rapid assessment of malaria transmission using age-specific sero-conversion rates. PLoS One. 2009;4(6):e6083. Available from: http://dx.doi.org/10.1371/journal.pone.0006083.

[5] Cook J, Reid H, Iavro J, Kuwahata M, Taleo G, Clements A, et al. Using serological measures to monitor changes in malaria transmission in Vanuatu. Malar J. 2010;9:169. Available from: http://dx.doi.org/10.1186/1475-2875-9-169.

[6] Cook J, Kleinschmidt I, Schwabe C, Nseng G, Bousema T, Corran PH, et al. Serological markers suggest heterogeneity of effectiveness of malaria control interventions on Bioko Island, equatorial Guinea. PLoS One. 2011;6(9):e25137. Available from: http://dx.doi.org/10.1371/journal.pone.0025137.

[7] Cook J, Speybroeck N, Sochanta T, Somony H, Sokny M, Claes F, et al. Sero-epidemiological evaluation of changes in Plasmodium falciparum and Plasmodium vivax transmission patterns over the rainy season in Cambodia. Malar J. 2012;11:86. Available from: http://dx.doi.org/10.1186/1475-2875-11-86.

[8] Hastie T, Tibshirani R, Friedman J. The Elements of Statistical Learning. 2nd ed. New York: Springer; 2009.

[9] Dudoit S, van der Laan MJ. Asymptotics of cross-validated risk estimation in estimator selection and performance assessment. Stat Methodol. 2005;2(2):131–154.

[10] Williams BG, Dye C. Maximum likelihood for parasitologists. Parasitol Today. 1994 Dec;10(12):489–493.
